# The effect of kinesiophobia on gastrointestinal disorders in patients with lower extremity orthopedic surgery

**DOI:** 10.3389/fsurg.2025.1457474

**Published:** 2025-04-25

**Authors:** Huseyin Gunes, Semra Bulbuloglu, Serdar Saritas, Ahmet Ozdemir

**Affiliations:** ^1^ Division of Surgical Nursing, Nursing Department, Faculty of Health Sciences, Bayburt University, Bayburt, Türkiye; ^2^ Division of Surgical Nursing, Nursing Department, Faculty of Health Sciences, Istanbul Aydın University, Istanbul, Türkiye; ^3^ Department of Medical Biology, Faculty of Medicine, Malatya Turgut Özal University, Malatya, Türkiye; ^4^ Division of Surgical Nursing, Nursing Department, Faculty of Health Sciences, Kahramanmaraş Sütçü İmam University, Kahramanmaraş, Türkiye

**Keywords:** fear of moving, gastrointestinal disorders, GI symptoms, kinesiophobia, lower extremity, orthopedic surgery, postoperative care

## Abstract

**Background:**

Postoperative kinesiophobia and gastrointestinal (GI) disorders are common and undesirable conditions following orthopedic surgery. Additionally, managing both conditions is crucial for preventing complications and accelerating recovery. The purpose of this study is to investigate the effects of kinesiophobia on GI disorders after lower extremity orthopedic surgery.

**Method:**

This study was conducted with a descriptive and cross-sectional design. The sample consisted of a total of *n* = 299 patients who underwent orthopedic surgery in their lower extremities at the orthopedics and traumatology clinic of a research and training hospital located in Turkey. A personal information form, the Tampa Scale of Kinesiophobia (TSK), and the Gastrointestinal Symptom Rating Scale (GSRS) were used to collect data, and the obtained data were analyzed using descriptive statistics, one-way analysis of variance (ANOVA), paired-samples t-test, and *post hoc* tests when necessary.

**Results:**

While 24.4% of the patients were aged 65–74 years, 51.5% were male. The mean total TSK score of the patients was above average (49.36 ± 8.74), while their mean total GSRS score was below average (31.22 ± 11.7). In our study, as kinesiophobia increased, the frequency of bowel movements decreased, and kinesiophobia explained 19.9% of the variance in GI disorders (*p* < 0.05).

**Conclusions:**

Kinesiophobia is a significant predictor of GI disorders in patients who underwent lower extremity surgery. Returning to normal GI function after surgery is crucial for preventing complications in patients with lower extremity surgery. Uncontrolled kinesiophobia after surgery exacerbates GI disorders. Therefore, early diagnosis and management of both kinesiophobia and GI disorders are necessary for rapid recovery in patients with lower extremity surgery.

## Introduction

Orthopedic surgery is considered the most intense procedure in terms of perceived pain during the postoperative period (1). Postoperative pain can lead patients to have anxiety and functional loss, causing kinesiophobia, where they become trapped in a vicious cycle and are unable to overcome this condition (2, 3). Kinesiophobia is an irrational fear that develops resistance to movement as a result of negative emotions such as pain or re-injury (4, 5). It is a psychological response to avoiding the fear of movement (6). While the fear experienced in the initial days after surgery to promote mobility of the operated limb is considered a natural phenomenon, it should be resolved as soon as possible because unresolved fear can turn into chronic kinesiophobia (7).

The incidence of kinesiophobia in orthopedic surgical patients has been reported to reach up to 52.8% (8). The patients who cannot cope with kinesiophobia and exhibit avoidance behavior towards movement may experience physical, physiological, and psychological problems such as depression, obsession, and lack of self-confidence (9–11). In the postoperative period, insufficient mobilization often leads to gastrointestinal (GI) disorders such as abdominal distention and constipation due to venous stasis and thrombus development, urinary retention, tissue integrity impairment, and slowing of peristalsis (12).

A previous study reported that 57.9% of the patients with orthopedic surgery experienced constipation in the period after their operation (13). The effect of early mobilization on GI functions is well-known, and surgical nurses have a responsibility to assess the GI system and perform effective interventions (14, 15). Various diseases have been reported to be associated with kinesiophobia in previous studies, including muscle and bone diseases and Parkinson's, as well as older people suffering from low back pain (16–19). Patients who have had lower extremity surgery may not be able to go to the toilet easily, so they need to ask for help from their companion or nurse. The patients may be embarrassed to say that they want to go to the toilet, which can result in constipation, indigestion, and ileus. The use of antibiotics and other medications after surgery can cause diarrhea. Other possible GI disorders in this patient group are not fully known. This study aimed to examine kinesiophobia and GI disorders in patients who have had lower extremity orthopedic surgery.

The hypotheses of this study are as follows:

H1: There are kinesiophobia and GI disorders in patients who received lower extremity orthopedic surgeries.
H0: Kinesiophobia and GI disorders are not associated with lower extremity orthopedic surgeries.

## Materials and methods

### Design and sample

This study had a descriptive and cross-sectional research method. The sample of the study consisted of patients who underwent lower extremity surgeries at the orthopedics and traumatology unit of a research and training hospital in eastern Turkey. A power analysis was performed for sample calculation, assuming a 0.05 margin of error and a 95% confidence interval, and a minimum of *n* = 196 patients were required to participate in the study. Due to possible data loss, this study included a total of *n* = 299 patients with lower extremity orthopedic surgery.

### Inclusion and exclusion criteria

The following criteria were determined for the inclusion of patients in this study (i) having undergone lower extremity surgery at the orthopedics and traumatology unit of the hospital and being on the 2nd, 3rd or 4th postoperative day, (ii) being aged 18 years and above without any communication problems, (iii) providing consent to take part in the study; (iv) having no diagnosed GI disorders/diseases before the surgery. Conditions contradicting the inclusion criteria were considered to be the exclusion criteria. In addition, those with GI disorders before surgery, preoperative disability and permanent physical disabilities, lumbar disc herniation, Parkinson's disease, or other neurological diseases were excluded.

### Data collection

The data were collected prospectively by the researchers in face-to-face interviews between February 1, 2022, and August 1, 2022. Each patient provided informed consent in written and verbal form. For patients who were illiterate, the data collection forms were read by the researchers, and the responses of the patients were marked on the form. Patients who were literate filled out the data collection forms by themselves. The data collection process was conducted in patient rooms and took an average of 30 min. Patients who completed 48 h postoperatively (between 48 and 96 h) and were allowed to mobilize by the physician were included in the sample.

### Data collection instruments

Data were obtained using a personal information form, the Tampa Scale of Kinesiophobia (TSK), and the Gastrointestinal Symptom Rating Scale (GSRS).

### Personal information form

The personal information form was developed by the researchers with expert consultation. It included questions designed to collect information on the socio-demographic characteristics of the patients (age, sex, marital status, occupation, education and income level), and health-related questions.

### Gastrointestinal symptom rating scale (GSRS)

The GSRS was created by Revicki et al. (1998) to assess commonly occurring symptoms in gastrointestinal conditions (20). The validity and reliability study of the scale in Turkish was conducted by Turan et al. (2017) (21). The GSRS measures how patients have been feeling in the context of GI disorders in the past week. It consists of 15 items and 5 subscales, namely reflux (2 items), dyspepsia (4 items), diarrhea (3 items), constipation (3 items), and abdominal pain (3 items). Higher GSRS scores show a higher severity of the relevant disorders. Turan et al. reported the Cronbach's alpha internal consistency coefficient of the GSRS to be 0.82. In this study, the Cronbach's alpha coefficient of the scale was determined to be 0.81.

### Tampa Scale for Kinesiophobia (TSK)

The TSK was developed by Swinkels-Meewisse et al. (2003) (22). The validity and reliability tests of the TSK in Turkish were performed by Yılmaz et al. (2011) (23). The TSK has 17 items to measure fear of movement, including 4 reverse-scored items (4, 8, 12, and 16). It includes parameters that detect fear or avoidance of injury/reinjury related to work-related activities. It uses a 4-point Likert-type scoring system (1 = strongly disagree, 4 = strongly agree), and its total score varies between 17 and 68. Higher total scores indicate higher levels of kinesiophobia (23).

### Statistical analysis

The data that were collected in this study were analyzed using the Statistical Package for the Social Sciences (SPSS) 25.0 IBM (Armonk, NY), and the evaluations included the calculation of descriptive statistics. Prior to the analysis, the Kolmogorov–Smirnov test was utilized to test whether the data were normally distributed. The one-way analysis of variance (ANOVA) and paired-samples t-test methods were used to identify the differences between the scale scores of the patients based on their descriptive characteristics. The Cronbach's alpha internal consistency coefficient, which is used as a measure of reliability, was calculated for the scales. *Post hoc* analyses were performed to determine the source of differences identified in ANOVA. Linear regression analysis was conducted to determine the predictors of variables among the scale total scores. Pearson correlation test was used to compare the scores of the scale and sub-dimensions. The results of the analyses were interpreted withing a 95% confidence interval and at a significance level of *p* < 0.05.

### Ethical considerations

For conducting the study, necessary legal permissions were received from the Institutional Review Board (IRB) of Turgut Özal Medical Center and the Ethics Committee of İnönü University (Date: 11.01.2022, Decision No: 2022/2954, Number: 01). In accordance with the Declaration of Helsinki, the researcher informed the patients about the study. Those who agreed to take part in the study were included after they provided verbal and written consent.

## Results

Table 1 presents the sociodemographic characteristics of the patients, as well as their GSRS and TSK mean scores, parametric test results, and *post hoc* test results. Of the patients, 24.4% were aged between 65 and 74, 51.5% were men, and 79.6% were married. In addition, 20.7% of the patients had tibia fracture surgery, 15.4% had total hip arthroplasty, and 11.4% had femur fracture repair. The percentage of patients who had one bowel movement per day before lower extremity surgery was 70.9%, which decreased to 38.5% after the surgery. The patients aged 75 years and above were more kinesiophobic compared to other age groups, and the retirees were more kinesiophobic compared to other occupational groups. As the TSK scores increased, the frequency of stool after surgery decreased, and this relationship was determined be significant.

**Table 1:** Sociodemographic characteristics, GSRS and TSK mean scores, parametric and *post hoc* tests results for patients with lower extremity surgery (*n* = 299).

Sociodemographic characteristics	*n* (%)	GSRS	TSK
Age			
Between 18 and 35 years (1)	67 (22.4)	30.90 ± 11.23	48.17 ± 8.13
Between 36 and 50 years (2)	58 (19.4)	30.87 ± 12.22	50.81 ± 7.13
Between 51 and 64 years (3)	68 (22.7)	30.58 ± 12.25	46.6 ± 8.52
Between 65 and 74 years (4)	73 (24.4)	33.33 ± 12.61	49.87 ± 9.85
75 years and above (5)	33 (11)	28.82 ± 7.06	53.75 ± 8.45
Test and value		*F* = 0.995, *p* = 0.490	*F* = 1.465, ***p*** **=** 0.046*
*Post hoc*			5 > 1,2,3,4
Gender			
Female	145 (48.5)	31.85 ± 11.7	50.44 ± 8.87
Male	154 (51.5)	30.65 ± 11.7	48.34 ± 8.53
Test and value		*t* = 0.964, *p* = 0.544	*t* = 1.633, ***p*** **=** 0.015*
Marital status			
Single	61 (20.4)	31.2 ± 11.02	48.27 ± 8.24
Married	238 (79.6)	31.22 ± 11.89	49.63 ± 8.86
Test and value		*t* = 0.972, *p* = 0.530	*t* = 0.975, *p* = 0.518
Educational level			
Literate	53 (17.7)	30.78 ± 7.92	51.58 ± 9.1
Primary school	109 (36.5)	31.47 ± 13.04	49.98 ± 9.1
High school	94 (31.4)	30.16 ± 9.85	47.9 ± 8.11
University and above	43 (14.4)	33.27 ± 14.84	48.23 ± 8.25
Test and value		*F* = 1.079, *p* = 0.348	*F* = 1.618, *p* = 0.116
Previous lower extremity surgery			
Tibia fracture surgery	62 (20.7)	30.35 ± 12.43	48.62 ± 7.49
Total knee arthroplasty	27 (9)	36.5 ± 16.04	53.45 ± 5.86
Total hip arthroplasty	46 (15.4)	39.93 ± 14.69	57.05 ± 6.42
Pelvic fracture repair	29 (9.7)	34.16 ± 10.33	56.96 ± 4.82
Femur fracture repair	34 (11.4)	31.52 ± 10.86	51.41 ± 7.99
Tendon repair	23 (7.7)	27.45 ± 7.77	46.45 ± 6.5
Other surgeries (Meniscus, patella fracture, hallux valgus, penetrating body injuries, ankle fracture etc.)	78 (26.1)	34.4 ± 8.14	53.40 ± 4.77
Test and value		*F* = 1.296, *p* = 0.107	*F* = 1.307, *p* = 0.118
Income level			
High income (1)	37 (12.4)	30.4 ± 12.04	48.89 ± 8.88
Middle income (2)	208 (69.6)	30.25 ± 10.78	48.47 ± 8.5
Low income (3)	54 (18.1)	35.51 ± 13.93	53.09 ± 8.77
Test and value		*F* = 1.543, ***p*** **=** 0.019*	*F* = 1.104, *p* = 0.320
*Post hoc*		3 > 1,2	
Occupation			
Housewife (1)	120 (40.1)	32.31 ± 12.31	51.15 ± 8.66
Worker (2)	52 (17.4)	29.73 ± 8.92	49.15 ± 6.39
Officer (3)	30 (10)	33.53 ± 15.81	48.83 ± 7.65
Self-employed (4)	39 (13)	31.44 ± 11.23	50.25 ± 9.1
Retired (5)	58 (19.4)	28.98 ± 10.22	51.89 ± 7.23
Test and value		*F* = 0.827, *p* = 0.783	*F* = 1.777, ***p*** **=** 0.005**
*Post hoc*			5 > 1,2,3,4
Frequency of stool after surgery			
1–3 times a day (1)	32 (10.7)	27.69 ± 10.15	45.78 ± 8.22
Once a day (2)	115 (38.5)	29.31 ± 8.48	45.53 ± 7.9
Once every two days (3)	89 (29.8)	30.56 ± 10.46	50.56 ± 8.47
Once every three days (4)	51 (17.1)	38.32 ± 13.12	55.29 ± 5.6
Once every four days (5)	12 (4)	46.45 ± 13.1	59.75 ± 3.1
Test and value		*F* = 2.161, ***p*** **=** 0.000**	F = 2.915, ***p*** **=** 0.000**
*Post hoc*		4,5 > 3 > 1,2	4,5 > 3 > 1,2

*F*, One-way analysis of variance ANOVA; *t*, Paired Sample t test.

* *p* < 0.05.

** *p* < 0.01.

Table 2 shows the patients’ TSK and GSRS mean scores. Their TSK and GSRS total mean scores were 49.36 ± 8.74 and 31.22 ± 11.7, respectively. Their GSRS subscales mean scores were as follows: Reflux 4.2 ± 2.71, Dyspepsia 8.36 ± 4.19, Diarrhea 4.51 ± 2.79, Constipation 6.76 ± 4.48, and Abdominal Pain 7.38 ± 3.27.

**Table 2:** TSK and GSRS mean scores (*n* = 299).

Total scale and subscales	Item number	Items	Score range	Min.–Max.	Xx ± SD
TSK total	17	1–17	17–68	27–67	49.36 ± 8.74
GSRS total	1–15	Items 1–15	15–105	15–80	31.22 ± 11.7
Reflux	2	Items 2 and 3	2–14	2–13	4.2 ± 2.71
Dyspepsia	4	Items 6, 7, 8 and 9	3–28	4–25	8.36 ± 4.19
Diarrhea	3	Items 11, 12 and 14	3–21	3–21	4.51 ± 2.79
Constipation	3	Items 10, 13 and 15	3–21	3–21	6.76 ± 4.48
Abdominal pain	3	Items 1, 4, and 5	3–21	3–18	7.38 ± 3.27

Table 3 displays the results of the linear regression analysis including the TSK and GSRS scores of the patients. According to these results, kinesiophobia explained 19.9% of the variance in GI disorders (R-squared = 0.199).

**Table 3:** Regression analysis between TSK and GSRS (*n* = 299).

Coefficients						
Model	Unstandardized coefficients		Standardized coefficients			
	B	Std. error	*F*	Beta	*t*	Sig.
1	(Constant)	1.774	3.540	-	0.501	0.617
	TSK	0.598	0.071	0.446	8.446	0.000^**^
Dependent variable: GSRS						
Model summary						
Model		R	R square	Adjusted R square		Std. error of the estimate
1		0.446	0.199	0.196		10.49
Predictors: (Constant), TSK						

^**^ *p* < 0.01.

Table 4 shows the correlation analysis between TSK and GSRS. According to the correlation analysis, there was a positive, strong and statistically significant correlation between kinesiophobia and constipation/abdominal pain (*p* < 0.001). There was a positive, moderate and statistically significant correlation between the total scores of TSK and GSRS (*p* < 0.05).

**Table 4:** Correlation analysis between TSK and GSRS (*n* = 299).

Total scale and subscales	TSK total	
	*r*	*p*
GSRS total	0.588	<0.05*
Reflux	0.505	0.654
Dyspepsia	0.349	0.207
Diarrhea	0.471	0.916
Constipation	0.228	<0.001**
Abdominal pain	0.232	<0.001**

*r*, Pearson Correlation test.

* *p* < 0.05.

** *p* < 0.01.

## Discussion

Kinesiophobia and GI disorders were examined in patients who have had lower extremity orthopedic surgery in this study. Age and gender affected the level of kinesiophobia. Since retired people are generally older people, the effect of employment status on kinesiophobia is an indirect effect. The relationship between kinesiophobia and frequency of stool after surgery was quite interesting, those who had more stool output were less kinesiophobic. Having a stool with someone's help or using a bedpan in bed is a very difficult experience. Patients may have preferred to go to the toilet alone rather than experience this difficulty and may have reduced their kinesiophobia. Every surgical procedure involves a controlled injury. Patients experience postoperative pain associated with surgical incisions. Although postoperative pain can be managed, it is often an inevitable experience (24, 25). Most of the time, patients avoid moving during the postoperative period due to pain. Patients who undergo lower extremity orthopedic surgery experience a high level of kinesiophobia in the early days after surgery (26). Kinesiophobia can lead to several problems in various body systems (12). In this context, kinesiophobia can slow down GI peristalsis and trigger various issues, including constipation (13, 14). From this perspective, it is very important to detect kinesiophobia and GI disorders in patients with lower extremity orthopedic surgery.

In this study, 24.4% of the patients were 65–74 years old, 51.5% were men, 79.6% were married, and 20.7% had tibia fracture surgery. Additionally, the patients aged 75 years and above were more kinesiophobic compared to other age groups, and the retirees were more kinesiophobic compared to other occupational groups. De Vroey et al. found kinesiophobia in patients who underwent lower extremity orthopedic surgery (27). Değirmenci et al. emphasized the development of kinesiophobia due to anesthesia in patients who underwent hip arthroplasty (26). Particularly in the elderly, transitioning to a less active lifestyle and increase in the incidence of falls may cause them to have kinesiophobia after lower extremity orthopedic surgery. The results related to kinesiophobia in the sample in this study are similar to the results of other studies in the literature.

According to these results, kinesiophobia is a predictor for the development of GI disorders, in this context it explained 19.9% of the variance in GI disorders. At the same time, the correlation analysis draws attention to the strong and positive correlation between kinesiophobia and constipation/abdominal pain. Park et al. reported a finding that was similar to ours, stating that the frequency of bowel movements decreased by half after surgery. They also indicated that patients with kinesiophobia and constipation had impaired adaptation to daily life activities (13). The results that were found in our study were in agreement with those in the relevant literature.

Early ambulation and mobilization are of great importance for the return of gastrointestinal activities during the postoperative period (28, 29). Approaches implemented within the Enhanced Recovery After Surgery (ERAS) protocol can eliminate the negative effects of stress responses in surgical patients and enable them to quickly transition to the discharge process. However, kinesiophobic patients tend to hinder this positive progress (14, 30). In the ERAS protocol, it is recommended for surgical patients to mobilize within 24 h at the latest and spend 2 h outside the bed on the day of surgery and 6 h on the following days. Kinesiophobia is one of the important obstacles to postoperative mobilization and can prolong morbidity by triggering GI disorders. Physicians do not want to discharge patients who are not well mobilized, and the treatment and kinesiophobia can reduce compliance of care and treatment.

A statistically significant and moderate level of correlation was identified between kinesiophobic behaviors and GI disorders in this study. Altay and Celenay found a statistically significant relationship between kinesiophobia and GI disorders in patients with migraines (31). Previous studies have noted that kinesiophobia resulted in a slowdown in daily life activities and functions in patients who underwent lower extremity orthopedic surgery (32), decreased their recovery speed (27, 33), and increased their perceived surgical pain (34). Reluctance to mobilize due to kinesiophobia, combined with slow blood circulation caused by anesthesia and physical energy deficiency, can lead to a slowdown in not only GI peristalsis but also other body system functions. This can cause various problems such as atelectasis, deep vein thrombosis, delayed wound healing, and increased stress response (14). Other predictors of postoperative GI disorders include various medication uses (35–37), depression (38), inability to relax (34), surgical pain (24), and infection (39).

In the present study was identified that kinesiophobia as a significant predictor of GI disorders, and these results will increase awareness of kinesiophobia among clinicians. The results of our study are specific to our sample and may not be generalizable to the general public. GI disorders in the patients may be due to their dietary habits, and the patients were still using medications during the postoperative period, which could have triggered their GI disorders. The stress caused by the surgery may have accelerated GI peristalsis, which may have caused diarrhea, poor pain management, and triggered knesiophobia. Pain and stress were not defined in this study. All of these factors can be considered as limitations for our study.

## Conclusion

In patients with lower extremity surgery, GI disorders are influenced by kinesiophobia. Early initiation of GI peristalsis after surgery is of great importance in preventing possible complications. Similarly, preventing kinesiophobia can facilitate social integration and shorten the length of hospital stay. Uncontrolled kinesiophobia can increase GI peristalsis, thus encouraging clinicians to use medication methods to ensure peristalsis. This situation requires additional medication use. Therefore, in addition to preventive interventions against kinesiophobia (such as playing calming music, mindfulness interventions, etc.), non-pharmacological methods such as abdominal massage, sufficient dietary fiber intake, liquid diet, and bed mobility exercises should be used to eliminate GI disorders.

## Data Availability

The raw data supporting the conclusions of this article will be made available by the authors, without undue reservation.
